# The Impact of Androgen Deprivation Therapy on COVID-19 Illness in Men With Prostate Cancer

**DOI:** 10.1093/jncics/pkac035

**Published:** 2022-05-12

**Authors:** Neil J Shah, Vaibhav G Patel, Xiaobo Zhong, Luis Pina, Jessica E Hawley, Emily Lin, Benjamin A Gartrell, Victor Adorno Febles, David R Wise, Qian Qin, George Mellgard, Himanshu Joshi, Jones T Nauseef, David A Green, Panagiotis J Vlachostergios, Daniel H Kwon, Franklin Huang, Bobby Liaw, Scott Tagawa, Philip Kantoff, Michael J Morris, William K Oh

**Affiliations:** Department of Medicine, Memorial Sloan Kettering Cancer Center, New York, NY, USA; Department of Medicine, Weill Cornell Medical Center, New York, NY, USA; Department of Medicine, Icahn School of Medicine at Mount Sinai, New York, NY, USA; Department of Medicine, Icahn School of Medicine at Mount Sinai, New York, NY, USA; Department of Medicine, Columbia University Medical Center, New York, NY, USA; Department of Medicine, Columbia University Medical Center, New York, NY, USA; Department of Medicine, Montefiore Center for Cancer Care, Bronx, NY, USA; Department of Medicine, Montefiore Center for Cancer Care, Bronx, NY, USA; Department of Medicine, NYU Langone Medical Center, New York, NY, USA; Department of Medicine, NYU Langone Medical Center, New York, NY, USA; Department of Medicine, Icahn School of Medicine at Mount Sinai, New York, NY, USA; Department of Medicine, Icahn School of Medicine at Mount Sinai, New York, NY, USA; Department of Medicine, Icahn School of Medicine at Mount Sinai, New York, NY, USA; Department of Medicine, Weill Cornell Medical Center, New York, NY, USA; Department of Medicine, Weill Cornell Medical Center, New York, NY, USA; Department of Medicine, Weill Cornell Medical Center, New York, NY, USA; Department of Medicine, University of California, San Francisco, CA, USA; Department of Medicine, University of California, San Francisco, CA, USA; Department of Medicine, Icahn School of Medicine at Mount Sinai, New York, NY, USA; Department of Medicine, Weill Cornell Medical Center, New York, NY, USA; Department of Medicine, Memorial Sloan Kettering Cancer Center, New York, NY, USA; Department of Medicine, Weill Cornell Medical Center, New York, NY, USA; Department of Medicine, Memorial Sloan Kettering Cancer Center, New York, NY, USA; Department of Medicine, Weill Cornell Medical Center, New York, NY, USA; Department of Medicine, Icahn School of Medicine at Mount Sinai, New York, NY, USA

## Abstract

**Background:**

TMPRSS2, a cell surface protease regulated by androgens and commonly upregulated in prostate cancer (PCa), is a necessary component for SARS-CoV-2 viral entry into respiratory epithelial cells. Previous reports suggested a lower risk of SARS-CoV-2 among PCa patients on androgen deprivation therapy (ADT). However, the impact of ADT on severe COVID-19 illness is poorly understood.

**Methods:**

We performed a multicenter study across 7 US medical centers and evaluated patients with PCa and SARS-CoV-2 detected by polymerase-chain-reaction between March 1, 2020, and May 31, 2020. PCa patients were considered on ADT if they had received appropriate ADT treatment within 6 months of COVID-19 diagnosis. We used multivariable logistic and Cox proportional-hazard regression models for analysis. All statistical tests were 2-sided.

**Results:**

We identified 465 PCa patients (median age = 71 years) with a median follow-up of 60 days. Age, body mass index, cardiovascular comorbidity, and PCa clinical disease state adjusted overall survival (hazard ratio [HR] = 1.16, 95% confidence interval [CI] = 0.68 to 1.98, *P* = .59), hospitalization status (HR = 0.96, 95% CI = 0.52 to 1.77, *P* = .90), supplemental oxygenation (HR 1.14, 95% CI = 0.66 to 1.99, *P* = .64), and use of mechanical ventilation (HR = 0.81, 95% CI = 0.25 to 2.66, *P* = .73) were similar between ADT and non-ADT cohorts. Similarly, the addition of androgen receptor–directed therapy within 30 days of COVID-19 diagnosis to ADT vs ADT alone did not statistically significantly affect overall survival (androgen receptor–directed therapy: HR = 1.27, 95% CI = 0.69 to 2.32, *P* = .44).

**Conclusions:**

In this retrospective cohort of PCa patients, the use of ADT was not demonstrated to influence severe COVID-19 outcomes, as defined by hospitalization, supplemental oxygen use, or death. Age 70 years and older was statistically significantly associated with a higher risk of developing severe COVID-19 disease.

COVID-19, caused by the severe acute respiratory syndrome coronavirus 2 (SARS-CoV-2) virus, has accounted for more than 4.5 million deaths globally (1). Mass vaccination efforts are taking place worldwide to mitigate the incidence and mortality related to COVID-19. Despite these efforts, COVID-19–related morbidity and mortality remain a global issue. Several new therapies are now approved, including monoclonal antibodies, dexamethasone, and remdesivir (2,3). Although this has led to improved clinical outcomes, more effective treatment strategies are still needed to reduce the complications and mortality of this disease.

Over the past year, translational investigations have identified potential vulnerabilities of SARS-CoV-2. Of particular relevance, SARS-CoV-2 relies on the host cell surface expression of angiotensin-converting enzyme 2 and transmembrane serine proteases 2 (TMPRSS2) for cellular entry into the respiratory epithelium (4). Influenza virus similarly uses TMPRSS2 for activation and cellular access (5,6). TMPRSS2-deficient mice infected with SARS-CoV or MERS-CoV displayed decreased viral levels and had less related damage in respiratory cells (7). Furthermore, TMPRSS2 inhibitors reduced infection of primary lung cells by SARS-CoV-2 (4). These findings demonstrate the critical role of TMPRSS2 in the pathogenesis of SARS-CoV-2 and its potential as a therapeutic target.

TMPRSS2 is commonly expressed in prostate cancer (PCa) cells and is regulated by androgens (8,9). In addition to the prostate, TMPRSS2 is also expressed in respiratory tissues. Androgen receptor (AR) expression is frequently observed in human lung cancer tissues (10). In fact, TMPRSS2 levels are decreased in pulmonary tissues of castrated C57BL/6 mice and upregulated by testosterone exposure, suggesting that TMPRSS2 expression in lung tissues may be driven by AR signaling. Hence, androgen deprivation therapy (ADT) may hypothetically reduce TMPRSS2 expression, limiting SARS-CoV-2 cellular entry and preventing severe complications from COVID-19. A recent report from Alimonti et al. (11) demonstrated a lower infection rate in PCa patients on ADT than those not on ADT. A recent study by Schmidt et al. (12) noted no association between ADT and 30-day mortality among patients with PCa and COVID-19. Given that, there is conflicting evidence on whether ADT use is protective against severe COVID-19 illness (13-15).

Herein, we report on our observational study of all patients with COVID-19 and PCa at 7 US medical centers to determine the impact of ADT on COVID-19–related clinical outcomes. To our best knowledge, this is one of the larger studies to report the severity of COVID-19 in patients with PCa and evaluate the association of ADT use with their clinical course associated with COVID-19 infection.

## Methods

### Data Acquisition

In this multi-institutional, retrospective, observational study across 7 US academic medical centers, we identified patients with a known diagnosis of PCa and SARS-CoV-2 viral detection by reverse-transcriptase-polymerase-chain-reaction from March 1, 2020, to May 31, 2020. The follow-up period was until last data cutoff, which was August 31, 2021. The patients were identified from both inpatient and outpatient settings. We collected clinical data, including medical comorbidities, medications, PCa diagnosis and therapy, and COVID-19–related clinical outcomes. Specifically, we collected data on hospital admission, oxygen requirements, the maximal amount of oxygen requirements (if applicable), mechanical ventilation, maximal score on the World Health Organization (WHO) COVID-19 ordinal scale for clinical improvement (WHO Ordinal scale) (16,17), and death status, at last follow-up on or before May 31, 2020. An institutional review board approval was obtained (#20-1263 ISMMS) for the study.

### Statistical Analysis

We summarized the descriptive statistics of all the demographic-, disease-, and treatment-related variables for patients who did or did not receive ADT, separately, by median (range) for continuous variables (eg, age) and frequencies (percentage) for categorical variables (eg, self-reported race from electronic health records). We then compared the distributions of these variables between the groups with vs without ADT using a *t* test for continuous variables and a χ^2^ test for categorical variables.

For the primary outcome of overall survival (OS), we used the Kaplan-Meier (KM) method to estimate the survival probabilities for patients with and without ADT. The OS was calculated from date of COVID-19 diagnosis to death or last follow-up date. The univariate analysis and multivariable analysis were conducted by using Cox proportional hazard regression models. We first built a series of Cox regression models between each potential risk factor and the outcome of OS in univariate analysis. These models were followed by a multivariable model that included all the predictors of interest. Of note, race and ethnicity were not statistically significantly associated with OS outcomes on univariate analysis (Supplementary Table 1, available online) and thus were not included in the adjusted models. The proportional hazard assumption was evaluated by observing the KM curves and testing the scaled Schoenfeld residuals.

We used logistic regression models to detect the associations between the predictors and each outcome in the analysis for secondary outcomes, including oxygen use, need for hospitalization, mechanical ventilation use, and severe illness. The WHO ordinal scale rates severe COVID-19 illness on a scale of 0–8: 0, uninfected; 1, ambulatory with no limitation of activities; 2, ambulatory with limitation of activities; 3, hospitalized, no oxygen therapy; 4, hospitalized and required oxygen by mask or nasal prongs; 5, hospitalized and required noninvasive ventilation or high-flow oxygen; 6, hospitalized, requires intubation and mechanical ventilation; 7, ventilation plus organ support, pressors, renal replacement therapy, extracorporeal membrane oxygenation; and 8, death. We defined severe COVID-19 illness as a WHO ordinal score of 5-8.

The possible correlation between the outcomes of patients in the same hospital was handled by a generalized estimation equation. Similar to the primary outcome analysis, we first conducted univariate analysis by a series of univariate logistic regression models between each predictor and a particular outcome. We then built the multivariable model by including all the predictors of interest. All the tests were 2-sided under the statistical significance level of .05. We used statistical software SAS (SAS Institute, Cary, NC, USA) for data analysis.

## Results

### Patient Characteristics

We identified 465 patients with PCa, including 31.8% (N  = 148) actively receiving ADT at the time of COVID-19 diagnosis. The baseline characteristics are listed in Table 1. The mean ages of patients on active ADT treatment (ADT) and those who did not receive ADT (noADT) were 73 years and 72 years, respectively. Compared with the noADT group, the ADT cohort had higher rates of body mass index (BMI) ≥ 30 (ADT, 32.4% vs noADT, 25.2%, *P* = .009) and lower rates of cardiovascular comorbidity (ADT, 73.0% vs noADT, 86.1%, *P* < .001), defined as having 1 or more risk factors, including hypertension, diabetes mellitus, or coronary artery disease. In terms of PCa characteristics, the ADT cohort, compared with the noADT cohort, had higher frequency of high-risk Gleason score at diagnosis (43.2% vs 15.1%, *P* < .001) and higher rates of metastatic disease (68.9% vs 5.0%, *P* < .001) (8-10). Among the ADT group, the most common site of metastasis was bone (n = 93, 62.8%), followed by lymph nodes (n = 47, 31.8%) and lung (n = 7, 4.8%). The other commonly used PCa-directed systemic therapies among the ADT cohort were AR-directed therapy (n = 63, 42.6%), chemotherapy (n = 16, 10.8%), and immune checkpoint inhibitors (n = 6, 4.1%).

**Table 1.** Baseline characteristic of prostate cancer patients diagnosed with COVID-19

Baseline characteristics	ADT, No. (%)	No ADT, No. (%)	*P* ^a^
Total No.	148	317	
Age, y			
Median	73	72	
≥70	93 (62.8)	187 (59.0)	.43
<70	55 (37.1)	130 (41.0)	
Race			.96
Black	39 (26.4)	88 (27.8)	
Others^b^/unknown	45 (30.4)	91 (28.7)	
White	64 (43.2)	138 (43.5)	
Ethnicity			.89
Hispanic	37 (25.0)	73 (23)	
Non-Hispanic	102 (68.9)	227 (71.6)	
Unknown	9 (6.1)	20 (6.3)	
BMI			.009
<30 kg/m^2^	100 (67.6)	237 (74.8)	
≥30 kg/m^2^	41 (28)	80 (25.2)	
Unknown	7 (4.7)	55 (17.4)	
Cardiovascular comorbidity^c^	108 (73.0)	273 (86.1)	<.001
Gleason grade at diagnosis			<.001
Low risk (6)	6 (4.1)	78 (24.6)	
Intermediate (7)	30 (20.3)	110 (34.7)	
High risk (8-10)	64 (43.2)	48 (15.1)	
Unknown	48 (32.4)	81 (25.6)	
Prostate cancer disease state			<.001
Nonmetastatic^d^	46 (31.1)	301 (95.0)	
Metastatic	102 (68.9)	16 (5.0)	
Non-ADT systemic anticancer therapies			
AR-directed therapy	63 (42.6)	7 (2.2)	<.001
Chemotherapy	16 (10.8)	0 (0.0)	<.001
Immune checkpoint inhibitor	6 (5)	0 (0.0)	<.001

a *P* values using 2-sided χ^2^ test of statistical significance. ADT = androgen-deprivation therapy; AR = androgen receptor; BMI = body mass index.

b Asian, American Indian or Alaska Native, Native Hawaiian or Other Pacific Islander.

c Presence of 1 or more cardiovascular risk factors, including hypertension, diabetes, and coronary artery disease.

d Localized, locally advanced, biochemical recurrent, or unknown.

### COVID-19–Related Clinical Outcomes

#### Entire Cohort

**Figure 1.**
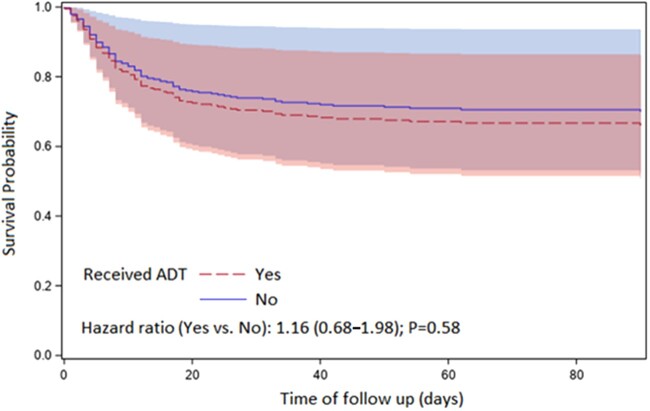
Overall survival (adjusted for age, body mass index, cardiovascular comorbidity, and clinical disease state) for prostate cancer patients diagnosed with COVID-19 receiving androgen-deprivation therapy (ADT) vs no ADT. The numbers within parentheses after the hazard ratio represent the 95% confidence interval.

The median follow-up period for the entire cohort was 60 (12-114) days. In the study population, 111 patients (24%) died due to COVID-19 illness. Table 2 describes the adjusted hazard ratio (HR) for each variable of interest, including ADT use, age, BMI, clinical disease state, and cardiovascular comorbidity. Gleason score was not included in adjusted analysis given 30% missing data. Compared with the noADT cohort, the ADT cohort had worse OS (HR 1.48, 95% CI = 1.01 to 2.17, *P* = .04). However, when adjusted for other variables of interest, OS was similar between the 2 groups (HR = 1.16, 95% CI = 0.68 to 1.98, *P* = .59) (Figure 1). Age was the only variable of interest that was statistically significantly associated with shorter OS. Specifically, older adults with age ≥70 years had shorter OS compared with younger men (HR = 3.45, 95% CI = 2.05 to 5.80, *P* < .001) when adjusted for the other variables. Furthermore, the presence of metastatic disease or cardiovascular comorbidities was not statistically significantly associated with worse OS (HR = 1.60, 95% CI = 0.93 to 2.75, *P* = .09; HR = 1.76, 95% CI = 0.91 to 3.38, *P* = .09, respectively).

**Table 2.** Overall survival outcome among prostate cancer patients diagnosed with COVID-19

Patient characteristics	Adjusted^a^ HR (95% CI)	*P* ^b^
ADT vs no ADT	1.16 (0.68 to 1.98)	.59
Age ≥70 vs <70 y	3.45 (2.05 to 5.80)	<.001
BMI ≥30 vs <30	0.93 (0.59 to 1.46)	.74
mPCa vs nmPCa	1.60 (0.93 to 2.75)	.09
CV risk factors^c^ vs none	1.76 (0.91 to 3.38)	.09

a Adjusted variable includes age, BMI, clinical disease state (localized vs metastatic) and cardiovascular risk factors. ADT = androgen-deprivation therapy; BMI = body mass index; CI = confidence interval; CV = cardiovascular; HR = hazard ratio; mPCa = metastatic prostate cancer; nmPCa = nonmetastatic prostate cancer (localized, locally advanced, or biochemically recurrent).

b Wald χ^2^ statistic (2-sided).

c One or more of the following cardiovascular risk factors: hypertension, diabetes, coronary artery disease.

We also evaluated other clinical outcomes, including illness severity, oxygen use, need for hospitalization, and requirement for mechanical ventilation (Table 3). When adjusted for age, BMI, PCa clinical disease state, and cardiovascular comorbidity, there were no statistically significant differences in these outcomes between patients receiving ADT and those not on ADT for hospitalization status (HR = 0.96, 95% CI = 0.52 to 1.77, *P* = .90), supplemental oxygenation (HR = 1.14, 95% CI = 0.66 to 1.99, *P* = .64), and use of mechanical ventilation (HR = 0.81, 95% CI = 0.25 to 2.66, *P* = .73), respectively. Of note, only age predicted severe illness, oxygen use, and hospitalization. Specifically, men aged 70 and older had more severe illness (HR = 1.64, 95% CI = 1.15 to 2.34, *P* = .006) and a higher likelihood of oxygen use (HR = 1.75, 95% CI = 1.21 to 2.54, *P* = .003) and hospitalization (HR = 2.18, 95% CI = 1.68 to 2.82, *P* < .001). However, there was no statistically significant difference in mechanical ventilation use (HR = 1.05, 95% CI = 0.76 to 1.46, *P* = .75).

**Table 3.** Clinical outcomes of COVID-19 illness among prostate cancer patients receiving ADT compared with no ADT

COVID-19 clinical outcomes	Adjusted^a^ HR (95% CI)	*P* ^b^
Overall survival	1.16 (0.68 to 1.98)	.59
Severe illness^c^	1.03 (0.57 to 1.87)	.91
Oxygen use	1.14 (0.66 to 1.99)	.64
Hospitalization	0.96 (0.52 to 1.77)	.90
Mechanical ventilation requirement	0.81 (0.25 to 2.66)	.73

a Adjusted for age, BMI, prostate cancer clinical disease state, cardiovascular comorbidity. ADT = androgen-deprivation therapy; BMI = body mass index; CI = confidence interval; HR = hazard ratio.

b Wald chi-square statistic (2-sided).

c Defined by World Health Organization Ordinal Scale for Clinical Improvement Score (5–8).

#### ADT-Only Cohort

In a subgroup analysis within the ADT cohort, we examined whether the addition of AR-directed therapy or chemotherapy, compared with ADT alone, affected COVID-19–related clinical outcomes. Again, these outcomes were adjusted for age. Of the 148 patients receiving ADT, 42.6% (n = 63) received concurrent AR-directed therapy and 10.8% (n = 16) received concurrent chemotherapy. Compared with patients on ADT alone, those receiving AR-directed treatment had similar OS (AR therapy: HR = 1.27, 95% CI = 0.69 to 2.32, *P* = .44). Similarly, other variables, including severe illness, oxygen use, hospitalization, and mechanical ventilation requirement, were not statistically significant (Table 4). Similarly, there were no statistically significant differences in COVID-19–related clinical outcomes between patients receiving ADT alone and those on ADT plus concurrent chemotherapy.

**Table 4.** Evaluating the impact of addition of ARDT or chemotherapy on COVID-19 clinical outcomes in patients receiving ADT

COVID-19 clinical outcomes	Unadjusted		Adjusted^a^	
	HR (95% CI)	*P* ^b^	HR (95% CI)	*P* ^b^
ARDT vs no ARDT				
Overall survival	1.52 (0.84 to 2.72)	.16	1.27 (0.69 to 2.32)	.44
Severe illness^c^	1.50 (0.66 to 3.41)	.34	1.28 (0.48 to 3.39)	.62
Oxygen use	1.77 (0.86 to 3.62)	.12	1.61 (0.70 to 3.70)	.27
Hospitalization	1.11 (0.59 to 2.08)	.75	1.00 (0.54 to 1.84)	>.99
Mechanical ventilation requirement	1.45 (1.19 to 1.78)	.003	1.14 (0.68 to 1.89)	.62
Chemo vs no chemo				
Overall survival	0.99 (0.39 to 2.51)	.98	1.06 (0.41 to 2.74)	.90
Severe illness^c^	1.51 (0.31 to 7.30)	.61	1.77 (0.38 to 8.38)	.47
Oxygen use	0.81 (0.35 to 1.88)	.62	0.80 (0.26 to 2.51)	.71
Hospitalization	1.27 (0.58 to 2.78)	.55	1.33 (0.60 to 2.90)	.48
Mechanical ventilation requirement	1.42 (0.30 to 6.79)	.66	1.19 (0.23 to 6.07)	.83

a Adjusted for age. ADT = androgen-deprivation therapy; AR = androgen receptor; ARDT = androgen receptor–directed therapy; CI = confidence interval; HR = hazard ratio.

b Wald χ^2^ statistic (2-sided).

c Defined by WHO Ordinal Scale for Clinical Improvement Score (5-8).

## Discussion

Over the past year, rapid innovation in vaccine and therapeutic development has reduced the morbidity and mortality related to COVID-19. However, the burden of the disease remains high worldwide. Discovering novel therapies remains a critical challenge. Understanding the virulence mechanisms of SARS-CoV-2 may provide implications for therapeutic and preventative methods to minimize the severity of this illness. Based on recent evidence, ADT could be a plausible effective strategy in men with COVID-19 (4,6). Yet, to date, robust retrospective and prospective clinical data are lacking regarding the clinical severity of COVID-19 in men receiving ADT. Because ADT is also the backbone of systemic therapy in PCa, we sought to study its potential effects on the clinical course of COVID-19 infection within a large, multi-institutional cohort of patients with PCa who tested positive for COVID-19 during the pandemic.

Preliminary studies focused on whether ADT could prevent the risk of acquiring COVID-19 infection. Montopoli et al. (11) were the first to report that infection rates were much lower in PCa patients receiving ADT than those not taking ADT. Furthermore, Caffo et al. (13), focusing only on patients with metastatic PCa on ADT, found that the risk of infection was higher than previously reported by Montopoli et al. (11). This may be related to men with advanced PCa being more vulnerable than patients with earlier stage PCa.

Here, we provide a more in-depth analysis of ADT use and the clinical severity of COVID-19 illness. First, our study suggests that ADT use did not influence survival, in line with findings from previous studies (12-15,17,18). Koskinen et al. (14), Kwon et al. (18), and Patel et al. (15) had further reported no statistically significant difference in the rate of infection and the need for mechanical ventilation between patients receiving ADT and those not receiving ADT (14,15). Similarly, Schmidt et al. (12) did not find any association between ADT and 30-day mortality among men with PCa. Our study complements these findings in a much larger and robust dataset. In addition, we noted similar hospitalization and oxygen use rates among PCa patients on active ADT vs noADT treatment.

We also sought to determine whether adding AR-directed therapy to ADT could have a protective effect against severe complications from COVID-19. In a preclinical PCa mouse model treated with enzalutamide, a novel antiandrogen, Gao et al. (19) noted distinct patterns of AR binding between prostate and lung epithelial cells. In addition, enzalutamide showed no antiviral activity against SARS-CoV-2 in mouse and human lung epithelial cells. Similarly, we did not find any statistically significant differences in patients receiving AR-directed therapy in conjunction with ADT compared with ADT alone. Currently, the ongoing COVIDENZA study (NCT04456049) is evaluating the efficacy of enzalutamide in high-risk men with COVID-19.

Another clinically valuable finding of our study was that age 70 years and older was statistically significantly associated with worse COVID-19 outcomes, including increased mortality, greater need for oxygen, and a higher chance of hospitalization. This is consistent with previous observations and provides additional evidence supporting the current public health strategy to protect this specific population (20-22). Other clinical variables, including race, ethnicity, smoking status, cardiovascular comorbidities, or concurrent medication, were not statistically significantly associated with clinical outcomes from COVID-19 illness.

Our study has several limitations. First, use of COVID-19–directed therapies may influence the severity of COVID-19 illness. During the study period, COVID-19–directed treatments were still evolving, rendering the recording of clinical information difficult. Secondly, other factors, including fear of testing, access to testing, access to a health-care facility, and local-regional prevalence of COVID-19, may have influenced the incidence and severity of COVID-19 in our study population. Finally, despite being a large study, our sample size was still limited, especially for the ADT plus chemotherapy cohort, and a larger dataset and/or prospective data would be necessary to fully address our question.

We report the largest study of COVID-19–related clinical outcomes after COVID-19 infection in PCa patients. We did not observe a statistically significant association between treatment with ADT and severity of COVID-19 illness in our study population. However, age 70 years and older was statistically significantly associated with a higher risk of developing severe COVID-19 disease and mortality. Prospective clinical trials with correlative science are warranted to answer this question more definitively.

## Funding

This research was funded in part through the National Institute of Health (NIH) / National Cancer Institute (NCI) Cancer Center Support Grant P30 CA008748.

## Notes

**Role of the funder:** The design, interpretation, and analysis of this study, the writing of the manuscript, and decision to submit the manuscript for publication rest solely with the authors.

**Disclosures: NJS:** Merk & Co. Inc.—Consultant, Database project; Aravive—Institutional funding for research and clinical trial support; **VP**: Seagen—Scientific Advisory Board; All funding or payments received are outside the scope of this study. **JH**: Regeneron, Denderon—Institutional Funding and research support; Genzyme—Trave and accommodation expenses. **DRW:** Pfizer, Janssen, Leap Therapeutics, Foundation Medicine, GLG, Guidepoint, Aptitude Health, Alphasights, Silverlight—Consultant; Pfizer—Travel; **ST:** Consulting or Advisory Role—4D Pharma; Abbvie; AIkido Pharma; Amgen; Astellas Pharma; Bayer; Blue Earth Diagnostics; Clovis Oncology; Dendreon; Endocyte; Genentech; Genomic Health; Immunomedics; Janssen; Karyopharm Therapeutics; Medivation; Novartis; Pfizer; POINT Biopharma; QED Therapeutics; Sanofi; Seattle Genetics; Tolmar Research Funding—Abbvie (Inst); Amgen (Inst); Astellas Pharma (Inst); AstraZeneca (Inst); AVEO (Inst); Bayer (Inst); Boehringer Ingelheim (Inst); Bristol-Myers Squibb (Inst); Clovis Oncology (Inst); Dendreon (Inst); Endocyte (Inst); Exelixis (Inst); Genentech (Inst); Immunomedics (Inst); Inovio Pharmaceuticals (Inst); Janssen (Inst); Karyopharm Therapeutics (Inst); Lilly (Inst); Medivation (Inst); Merck (Inst); Millennium (Inst); Newlink Genetics (Inst); Novartis (Inst); POINT Biopharma (Inst); Progenics (Inst); Rexahn Pharmaceuticals (Inst); Sanofi (Inst); Stem CentRx (Inst) Travel, Accommodations, Expenses—Amgen; Immunomedics; Sanofi; **PK:** Convergent Therapeutics, Context Therapeutics, XLink—Leadership, Convergent Therapeutics, Placon, Druggablity Technologies, Context Therapeutics, Seer, Cogent Biosciences, Mirati Therapeutics, PrognomIQ, SynDevRx, XLink—Stocks and Other Ownership Interest; Janssen, Merck, OncoCellMDX, Genentech/Roche, Tarveda Therapeutics, Druggablity Technologies, Progenity, Seer, Anji, Candel Therapeutics, Context Therapeutics, PrognomIQ, SynDevRx, Veru, XLink—Consulting or Advisory Role; All funding or payments received are outside the scope of this study; **MM:** Curium, Athenex, ORIC, and Exelexis—Consultant; Norvartis, Advanced Accelerator Applications, Progenics, Lantheus, Janssen, Endocyte, and Bayer—Uncompensated Consultant Bayer, Endocyte, Progenics, Corcept, Roche/Genentech, and Janssen—Institutional Funding for research and clinical trials WKO: Astellas, Astra-Zeneca, Bayer, Janssen, Pfizer and Sanofi—Consultant; Sema4-Chief Medical Science Officer; All funding or payments received are outside the scope of this study: **XZ, LP, EL, BAG, VAF, QQ, GM, HJ, JTN, DAG, PJV, DHK, FH, BL—**No conflicts of interest.

**Author contributions:** Conceptualization—WKO, MJM, PK, ST, BL, DHK, DAG, DRW, BAG, JEH, XZ, VGP and NJS. Investigation—NJS, VGP, LP, EL, VAF, QQ, GM, JTN, PJV, FH. Formal Analysis—XZ and HJ. Writing-original draft—NJS, VGP. Writing-review & editing—All authors.

**Prior presentations:** Poster presentation, American Society of Clinical Oncology Genitourinary Cancers Symposium, February 11th, 2021, San Francisco, USA. J Clin Oncol 39, 2021 (suppl 6; abstr 41).

## Data Availability

The raw data used for this analysis are not publicly available due to privacy or ethical restrictions. The deidentified data will be made available to appropriate personal upon request to corresponding author.

## Supplementary Material

pkac035_Supplementary_Data

## References

[1] https://www.worldometers.info/coronavirus/. Published 2021. Accessed May 28, 2021.

[2] Beigel JH , Tomashek KM, Dodd LE, et al Remdesivir for the treatment of Covid-19—final report. N Engl J Med. 2020;383(19):1813-1826. 32445440 10.1056/NEJMoa2007764 PMC7262788

[3] Horby P , Lim WS, Emberson J, et al Dexamethasone in hospitalized patients with Covid-19. N Engl J Med. 2021;384(8):693-704. 32678530 10.1056/NEJMoa2021436 PMC7383595

[4] Hoffmann M , Kleine-Weber H, Schroeder S, et al SARS-CoV-2 cell entry depends on ACE2 and TMPRSS2 and is blocked by a clinically proven protease inhibitor. Cell. 2020;181(2):271-280.e8. 32142651 10.1016/j.cell.2020.02.052 PMC7102627

[5] Chaipan C , Kobasa D, Bertram S, et al Proteolytic activation of the 1918 influenza virus hemagglutinin. J Virol. 2009;83(7):3200-3211. 19158246 10.1128/JVI.02205-08 PMC2655587

[6] Matsuyama S , Nagata N, Shirato K, Kawase M, Takeda M, Taguchi F. Efficient activation of the severe acute respiratory syndrome coronavirus spike protein by the transmembrane protease TMPRSS2. J Virol. 2010;84(24):12658-12664. 20926566 10.1128/JVI.01542-10 PMC3004351

[7] Iwata-Yoshikawa N , Okamura T, Shimizu Y, Hasegawa H, Takeda M, Nagata N. TMPRSS2 contributes to virus spread and immunopathology in the airways of murine models after coronavirus infection. J Virol. 2019;93(6):e01815-e01818. 30626688 10.1128/JVI.01815-18 PMC6401451

[8] Lin B , Ferguson C, White JT, et al Prostate-localized and androgen-regulated expression of the membrane-bound serine protease TMPRSS2. Cancer Res. 1999;59(17):4180-4184. 10485450

[9] Mostaghel EA , Nelson PS, Lange P, et al Targeted androgen pathway suppression in localized prostate cancer: a pilot study. J Clin Oncol. 2014;32(3):229-237. 24323034 10.1200/JCO.2012.48.6431 PMC3887479

[10] Mikkonen L , Pihlajamaa P, Sahu B, Zhang FP, Janne OA. Androgen receptor and androgen-dependent gene expression in lung. Mol Cell Endocrinol. 2010;317(1–2):14-24. 20035825 10.1016/j.mce.2009.12.022

[11] Montopoli M , Zumerle S, Vettor R, et al Androgen-deprivation therapies for prostate cancer and risk of infection by SARS-CoV-2: a population-based study (n=4532). Ann. Oncol. 2020;31(8):1040-1045. 32387456 10.1016/j.annonc.2020.04.479 PMC7202813

[12] Schmidt AL , Tucker MD, Bakouny Z, et al Association between androgen deprivation therapy and mortality among patients with prostate cancer and COVID-19. JAMA Netw Open. 2021;4(11):e2134330. 34767021 10.1001/jamanetworkopen.2021.34330 PMC8590166

[13] Caffo O , Zagonel V, Baldessari C, et al On the relationship between androgen-deprivation therapy for prostate cancer and risk of infection by SARS-CoV-2. Ann Oncol. 2020;31(10):1415-1416. 32562741 10.1016/j.annonc.2020.06.005 PMC7299865

[14] Koskinen M , Carpen O, Honkanen V, et al Androgen deprivation and SARS-CoV-2 in men with prostate cancer. Ann Oncol. 2020;31(10):1417-1418. 32615154 10.1016/j.annonc.2020.06.015 PMC7323668

[15] Patel VG , Zhong X, Liaw B, et al Does androgen deprivation therapy protect against severe complications from COVID-19? Ann Oncol. 2020;31(10):1419-1420. 32653425 10.1016/j.annonc.2020.06.023 PMC7347319

[16] World Health Organization. Ordinal scale. https://www.who.int/blueprint/priority-diseases/key-action/COVID-19_Treatment_Trial_Design_Master_Protocol_synopsis_Final_18022020.pdf. Accessed May 31, 2021.

[17] Klein EA , Li J, Milinovich A, et al Androgen deprivation therapy in men with prostate cancer does not affect risk of infection with SARS-CoV-2. J Urol. 2021;205(2):441-443. 32897764 10.1097/JU.0000000000001338

[18] Kwon DH , Vashisht R, Borno HT, et al Androgen-deprivation therapy and SARS-CoV-2 in men with prostate cancer: findings from the University of California Health System registry. Ann Oncol. 2021;32(5):678-679. 33571636 10.1016/j.annonc.2021.01.067 PMC7870099

[19] Li F , Han M, Dai P, et al Distinct mechanisms for TMPRSS2 expression explain organ-specific inhibition of SARS-CoV-2 infection by enzalutamide. Nat Commun. 2021;12(1):866. 33558541 10.1038/s41467-021-21171-x PMC7870838

[20] Richardson S , Hirsch JS, Narasimhan M, et al the Northwell COVID-19 Research Consortium. Presenting characteristics, comorbidities, and outcomes among 5700 patients hospitalized with COVID-19 in the New York City area. JAMA. 2020;323(20):2052-2059. 32320003 10.1001/jama.2020.6775 PMC7177629

[21] Williamson EJ , Walker AJ, Bhaskaran K, et al Factors associated with COVID-19-related death using OpenSAFELY. Nature. 2020;584(7821):430-436. 32640463 10.1038/s41586-020-2521-4 PMC7611074

[22] Wu Z , McGoogan JM. Characteristics of and important lessons from the coronavirus disease 2019 (COVID-19) outbreak in China: summary of a report of 72314 cases from the Chinese Center for Disease Control and Prevention. JAMA. 2020;323(13):1239-1242. 32091533 10.1001/jama.2020.2648

