# Supplementary material for: The Impact of Androgen Deprivation Therapy on COVID-19 Illness in Men With Prostate Cancer
Source: JNCI Cancer Spectr. 2022 May 12;6(3):pkac035. doi: 10.1093/jncics/pkac035 (PMC9165550; doi:10.1093/jncics/pkac035)
Supplement: pkac035_Supplementary_Data [file pkac035_supplementary_data.pdf]

SUPPLEMENTARY MATERIALS

Supplementary Table 1. Unadjusted analysis of overall survival among prostate cancer patients diagnosed with COVID-19

Patient Characteristics	Unadjusted HR (95% CI)	*P*[a]
ADT vs. no ADT	1.48 (1.01, 2.17)	0.04
Age $\geq$ 70 vs. <70 years	3.75 (2.29, 6.16)	<0.001
Race		
Black or African American	1.28 (0.82, 2.01)	0.28
Other[b]	1.21 (0.75, 1.95)	0.44
White	1.00 (Referent)	
Ethnicity		
Hispanic vs. Non-Hispanic	1.31 (0.85, 2.00)	0.22
BMI $\geq$ 30 vs. < 30 kg/m^2	0.86 (0.55, 1.34)	0.51
mPCa vs. nmPCa	1.73 (1.17, 2.57)	0.006
CV risk factors[c] vs. none	2.17 (1.17, 4.05)	0.02

[a] Wald chi-square statistic (2-sided). ADT = androgen deprivation therapy, BMI = body mass index, CI = confidence interval; CV = cardiovascular, HR = hazard ratio; mPCa = metastatic prostate cancer, nmPCa = non-metastatic prostate cancer (localized, locally advanced, or biochemically recurrent)

[b] Asian, American Indian or Alaska Native, Native Hawaiian or Other Pacific Islander.

[c] One or more of the following cardiovascular risk factors: Hypertension, Diabetes, Coronary artery disease
